# Formalin-induced and neuropathic pain altered time estimation in a temporal bisection task in rats

**DOI:** 10.1038/s41598-019-55168-w

**Published:** 2019-12-10

**Authors:** Xinhe Liu, Ning Wang, Jinyan Wang, Fei Luo

**Affiliations:** 1 CAS Key Laboratory of Mental Health, Institute of Psychology, Chinese Academy of Sciences, Beijing, 100101 P.R. China; 2 Department of Psychology, University of Chinese Academy of Sciences, Beijing, 100049 P.R. China

**Keywords:** Perception, Psychology

## Abstract

Time perception is an important ability that is related closely to humans’ and animals’ daily activities. It can be distorted by various emotional states. In human studies, experimental pain has been shown to prolong the perception of time. However, related animal studies are lacking. In this study, we used a temporal bisection task to investigate how acute inflammatory pain (induced by hind-paw formalin injection) and chronic neuropathic pain [induced by spinal nerve ligation (SNL)] affected time perception in rats. Rats were trained to recognize “short” (1200-ms) and “long” (2400-ms) anchor-duration pure tones and were rewarded for corresponding lever presses. During testing, rats perceived a series of intermediate-duration and anchor-duration pure tones, and selected levers corresponding to the “short” and “long” tones. After formalin injection, rats gave more “long” lever-press responses than after saline injection. The point of subjective equality after formalin injection also increased, suggesting that formalin-induced acute pain extended time perception. In contrast, rats that had undergone SNL gave fewer “long” lever-press responses compared with the sham surgery group. This animal study suggests that formalin-induced pain and neuropathic pain may have different effects on time perception.

## Introduction

Time estimation is a critical ability of animals and humans in their daily activities^1,2^. A leopard needs to choose the right time to attack its prey; a chef needs to estimate the cooking time to ensure that a dish has the best flavor. However, the subjective perception of time is not invariable; it depends on the environmental context and emotional state. Previous studies have suggested that time perception is regulated by many factors, including emotion, motivation, and attention^2–4^. When faced with a threat stimulus, such as an angry face^5,6^ or a frightening spider^7,8^, human subjects overestimate the amount of time passed. By contrast, in high approach motivation states, humans and animals perceive that time passes more quickly^4,9^. In addition, a series of studies showed that subjective time perception could be shortened when subjects were distracted from to-be-timed stimuli^10–12^.

Pain is a multidimensional subjective experience, with sensory-discriminative, affective-motivational, and cognitive-evaluational components^13^. It can affect psychological processes such as decision making^14,15^, attention^16,17^, and working memory^18^. Some human studies have also shown that time perception is affected by acute pain. Subjects have been found to overestimate the neutral visual stimulation time when feeling radiant heat pain^19^ and in a cold pressor test^20^. The influence of chronic pain on time perception is more complex. Many patients with chronic pain perceive that time passes more slowly in daily life^21,22^. In a study conducted with migraineurs, Anagnostou and Mitsikostas^23^ found that temporal estimation remained normal for most subjects, but was prolonged in a subgroup with depressive symptoms. The threshold of somatosensory time discrimination has also been found to be increased during migraine attacks^24,25^. However, animal studies of pain and time perception are lacking. Appropriate animal models will aid exploration of the neurological and pharmacological mechanisms of pain’s effects on time perception.

In this study, we assessed changes in time perception under acute inflammatory pain and chronic neuropathic pain in rats using a temporal bisection task. Formalin injection was used to induce acute inflammatory pain and spinal nerve ligation (SNL) was used to induce chronic neuropathic pain. The temporal bisection task is a widely accepted paradigm for the study of time perception in animals and humans^9,20,26–28^; we trained rats to discriminate “long” and “short” anchor durations of a pure tone, and to press corresponding levers. The psychometric function was used to fit the data obtained and to examine response bias and sensitivity^29,30^.

## Materials and Methods

### Animals

Male Sprague-Dawley rats (weight 230–250 g on arrival, 260–280 g before experiments; Charles River, Beijing, China) were used in this study. All rats were housed individually with food and water available ad libitum in a temperature- and humidity-controlled room (22°C, 65% humidity), maintained with a reverse 12:12-h light:dark cycle (light on at 8:00 pm). After arriving in the laboratory, the rats were adapted to the environment for at least 1 week. Before the experiments, they were handled daily to familiarize them with the manipulation of the experimenter. All the experimental procedures were approved by the Institutional Animal Care and Use Committee of Chinese Academy of Sciences. We confirm that all methods were performed in accordance with the relevant guidelines and regulations.

### Temporal bisection task

The training sessions and temporal bisection task were carried out in the same operant box (21 cm W × 32.5 cm D × 42.5 cm H), which was located in a dim soundproof chamber. The walls of the operant box were made of acrylic, and the floor was made of acrylic strips and positioned above a catch pan. Two retractable levers (ENV-112CMP; MED Associates, American) were mounted symmetrically on one side wall of the box (9 cm above the floor), with a liquid dispenser (ENV-201A; MED Associates, American) and water receptacle located between and equidistant from them (2.5 cm above the floor) to provide water as the reinforcer. A tone generator (ENV-223AM; MED Associates, American) mounted on the wall was used to present the pure tone stimuli (2900 Hz, 65 dB). On the middle of the opposite wall, an illuminated infrared detective nose-poke hole (ENV-114BM; MED Associates, American) was mounted 2.5 cm above the floor, and an indicator lamp (ENV-221M; MED Associates, American) was mounted directly above it. All output and input devices mounted in the operant box were furnished by the experimenters.

**Figure 1:**
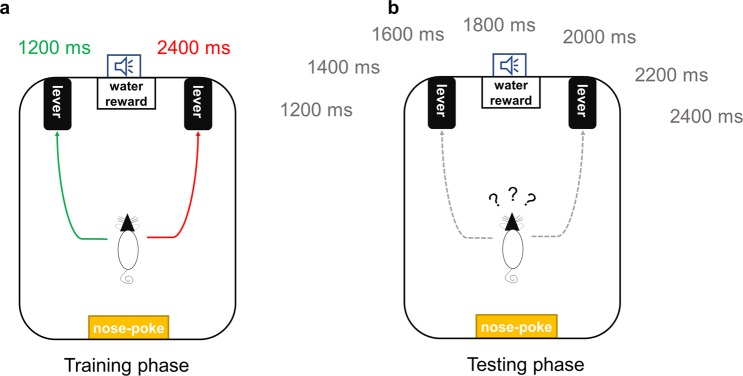
Overview of temporal bisection training and task. (**a**) During the training stage, rats were trained to discriminate two anchor-duration pure tones and to press the corresponding levers. (**b**) During the test stage, several intermediate-duration tones were presented as well as the anchor-duration tones, and rats were free to select which lever to press.

Temporal discrimination training was conducted using a previously described procedure^9,31,32^ with modification. Right and left lever-press responses following tones with anchor durations of 1200 and 2400 ms were reinforced (Fig. 1a). The relationship of the two anchor durations with the two levers was counterbalanced. In the last three free-choice training sessions, the accuracy of all rats’ responses to the anchor-duration tones exceeded 85% and the average accuracy in each group of rats exceeded 90%.

After discrimination training, the temporal bisection task was conducted (Fig. 1b). This task comprised 140 trials (30 s/trial; total duration, 70 min): 14 trials each with tones of five intermediate durations (1400, 1600, 1800, 2000, and 2200 ms) without reinforcement, and 35 trials each with the two anchor-duration tones (1200 and 2400 ms) with reinforcement. The order of trials was random, but with the constraint of no more than three consecutive intermediate-duration trials. Sessions began with illumination of the indicator light. The to-be-timed tone was initiated by activation of the nose-poke hole. Its termination was followed by immediate presentation of the two levers. In the anchor-duration trials, correct responses were reinforced with drips of water. For each test session, the trials were presented in the same order for all animals.

### Establishment of the pain model

Acute inflammatory pain was induced by injection of 50 μl 1% formalin solution into the hind paw. Saline injection was used as the control. Each rat was returned to the operant box immediately after injection, and the nociceptive behavior of the injected paw was recorded for 70 min. After the test stage, rats that received the formalin injection were placed in the operant box again without the temporal bisection task. We calculated the durations of lifting and licking of the injected paw in 5-min epochs.

L5 SNL was used to establish an animal model of neuropathic pain. According to the procedure of Kim and Chung^33^, rats were placed in a prone position and anesthetized with 50 mg/kg sodium pentobarbital (i.p). The fur on the left side of the spine was then shaved to expose the skin. The skin was cut parallel to the spine and the paraspinal muscles were separated. The L5 transverse process was removed until the L5 spinal nerve was exposed. The L5 nerve was isolated and ligated with silk thread. Finally, the muscle and skin incisions were sutured and the wound was disinfected with iodophor and 75% ethyl alcohol. In the sham group, rats underwent the same surgical procedure without the nerve ligation.

### Behavioral tests

The von Frey test with the up-down method described by Dixon *et al*.^34^ was used to measure the 50% paw withdrawal threshold (PWT) of rats in the neuropathic pain experiment. All rats were allowed to acclimatize to the environment for at least 10 min before the test. In brief, the rats were placed on an iron metal grid, restricted and separated with plastic covers. von Frey filament (0.008–15.0 g) was applied vertically to the plantar surfaces of the hind paw ipsilateral or contralateral to the SNL/sham surgery. The test position was on the rear hind paw (avoiding the pads), as described previously^35^. Sharp paw lifting within 4 s was defined as a positive response.

Radiant heat was used to examine thermal hyperalgesia of rats in the neuropathic pain experiment. As described in our previous studies^36^, the rats were placed on a transparent plexiglass plate and separated with plastic covers to restrict their activity. A radiant heat source beneath the floor was aimed at the plantar surface of the hind paw ipsilateral or contralateral to the SNL/sham surgery. In each test, the heat source was turned on until the rat quickly withdrew its hind paw or until the cutoff time of 25 s. Paw withdrawal latency (PWL) was defined as the time between heat onset and paw lifting. Five trials were conducted on each hind paw at 5-min intervals. PWL values from the last 3 trials were averaged.

### Experimental procedure

We adopted a within-subjects design to assess the effect of formalin-induced pain on time perception (*n* = 10). After the temporal discrimination training stage, all rats received two injections (one of saline and one of formalin) at a 7-day interval. To balance the order effect, half of the rats received saline injections first, and the other half received formalin injections first. The rats performed the bisection task immediately after saline/formalin injection.

Two groups of rats (SNL and sham; *n* = 10 each) were used to assess the effect of neuropathic pain on time perception in this experiment. The von Frey test and thermal paw withdrawal test were performed before surgery (baseline) and 14 days after surgery. Two days before surgery, the temporal bisection task was performed as a baseline measure. After surgery, the rats were allowed to recover for 7 days. On days 8–13, the rats underwent five training sessions to restore their temporal discrimination ability. On postoperative days 14 and 15, during the period of sensitivity to neuropathic pain^37–39^, the temporal bisection task was administered again (Once per day, to ensure the stability of the results).

### Data analysis

The proportion of long response (P_L_) to each tone duration in the temporal bisection task was used to assess time perception. The function related to P_L_ and the duration is a typically sigmoidal, as an example of psychometric function. Following widespread practice^29–31^, we adopted a sigmoidal cumulative Gaussian function to fit the experimental data from the temporal bisection tasks. This function can be expressed as:

1 $$f(t)=a+\frac{b}{\sqrt{2\pi \sigma }}[exp-(\frac{{(t-\mu )}^{2}}{2{\sigma }^{2}})],$$

where ƒ(t) represents the expected P_L_ when the duration is equal to a given sample t, *µ* represents the mean and *σ* represents the standard deviation (SD), *a* represents the low asymptote of the function, and *b* represents the range. The point of subjective equality (PSE), defined as a tone duration that animals have 50% chance of perceiving as “short” or “long,” is equal to *µ*. An increase or decrease in the PSE value is an index of the response bias to a “long” or “short” tone, which was interpreted generally as the subjective under- or overestimation of time^40^. The SD, equal to *σ*, represents the slope of the function and was calculated as a measure of temporal sensitivity. A decrease in the SD indicates temporal sensitivity to the durations increase^29–31^. Curve fitting and parameter calculation were performed using Prism software (version 5, GraphPad software, Inc).

For the temporal bisection task, trials with no lever-press response were omitted from the analysis. Three-way and two-way analyses of variance, and independent-sample and paired-sample *t* tests, were used with appropriate factors for data analysis. The Duncan test was adopted as a post-hoc test, and *p* < 0.05 was considered to indicate significance. All data are expressed as means ± standard errors of the mean. The statistical analyses were conducted with Prism (version 5, GraphPad software, Inc) and STATISTICA (version 6, StatSoft.Inc) software.

## Results

### Effect of formalin-induced acute inflammatory pain on time perception

**Figure 2:**
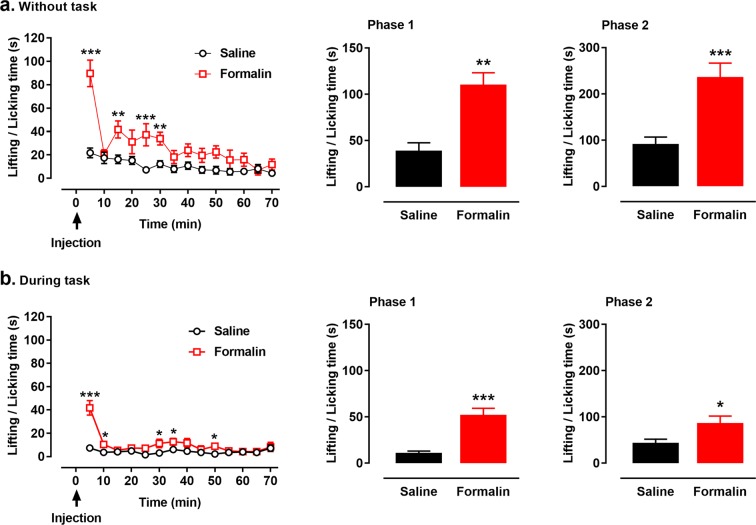
Nociceptive behaviors after formalin/saline injection during and without the temporal bisection task. Rats received single injections of 1% formalin solution (50 μl) into the hind paw. Nociceptive behavior was measured as the time spent in lifting and licking the injected paw, which varied as a function of time. Formalin injection induced more nociceptive behaviors during 70 min without the task (**a**) and during the task (**b**). Cumulative paw-licking scores in phases 1 (middle panel of a, b) and 2 (left panel of a, b) after formalin injection were significantly higher than these after saline injection. *p < 0.05, **p < 0.01, ***p < 0.001 vs. saline injection; *n* = 10. Results are shown as mea*n*s ± SEM.

A typical biphasic pattern of nociceptive behavior was observed after formalin injection into the hind paw. With no temporal bisection task, rats injected with formalin exhibited much more paw lifting and licking behavior than did rats injected with normal saline [interactive effect: *F*_(13, 117)_ = 5.211, *p* < 0.001; treatment effect: *F*_(1, 9)_ = 41.394, *p* < 0.001; time effect: *F*_(13, 117)_ = 12.331, *p* < 0.001; Fig. 2a, left panel]. The cumulative time spent in paw lifting and licking was significantly increased in phase 1 [0–10 min; *t*_(9)_ = 6.668, *p* = 0.001; Fig. 2a, middle panel] and phase 2 [20–60 min; *t*_(9)_ = 5.911, *p* < 0.001, Fig. 2a, right panel] after formalin injection compared with the time spent after saline injection.

Similarly, during the temporal bisection task, more nociceptive behavior was observed after formalin injection than after saline injection [interactive effect: *F*_(13, 117)_ = 8.212, *p* < 0.001; treatment effect: *F*_(1, 9)_ = 22.029, *p* = 0.001; time effect: *F*_(13, 117)_ = 10.371, *p* < 0.001; Fig. 2b, left panel]. Formalin injection significantly increased the cumulative time spent in paw lifting and licking in phase 1 [0–10 min; *t*_(9)_ = 4.663, *p* < 0.001; Fig. 2b, middle panel] and phase 2 [20–60 min; *t*_(9)_ = 2.836, *p* = 0.020; Fig. 2b, right panel] compared with that spent after saline injection. By comparing the cumulative time spent in paw lifting and licking in 0–70 min, less nociceptive behavior was observed during the temporal bisection task than without the task after formalin injection [during task vs. without task: 144.4 ± 57.0 s vs. 389.0 ± 122.7 s, *t*_*(9)*_ = 5.654, *p* < 0.001]. Besides, the cumulative time spent in paw lifting and licking after formalin injection during the task were significantly shorter than that without task [phase 1: *t*_(9)_ = 4.015, *p* = 0.003; phase 2: *t*_(9)_ = 5.015, *p* = 0.001].

**Figure 3:**
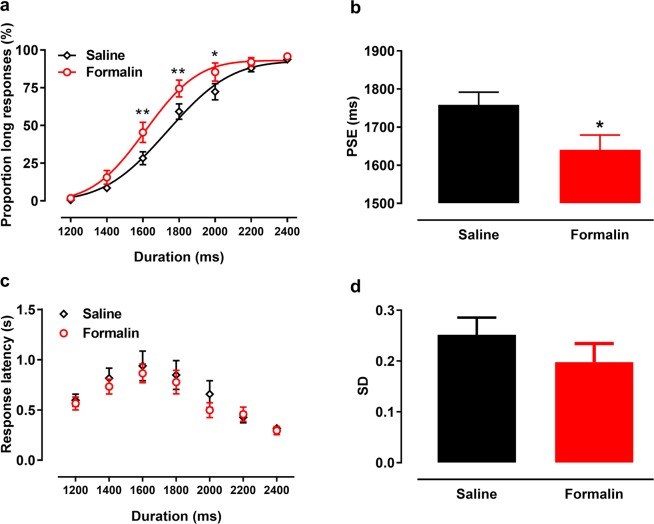
Response patterns in temporal bisection tasks after formalin/saline injection [Proportion of long response (P_L_) by duration and psychometric fitting curves]. (**a**) After formalin injection, the fitting curve shifted left compared with saline injection and baseline. (**b**) Formalin injection significantly decreased the point of subjective equality (PSE). Response latency (**c**) and the standard deviations (SD) of fitting function (**d**) did not differ between conditions. *p < 0.05, **p < 0.01 vs. saline injection; *n* = 10. Results are shown as means ± SEM.

During the test phase, P_L_s increased with increasing tone duration, forming nearly standard sigmoid curves (Fig. 3a). The goodness of fit of the Gaussian function for individual rats was ≥0.90. Under the effect of 1% formalin, the fitted curve shifted to the left and upward; rats gave more “long” anchor-duration lever-press responses after formalin injection than after saline injection [interactive effect: *F*_(6, 54)_ = 1.454, *p* = 0.212; treatment effect: *F*_(1, 9)_ = 6.186, *p* = 0.035; Fig. 3a]. Specifically, formalin injection increased the P_L_s for 1600-ms (*p* = 0.003), 1800-ms (*p* = 0.009), and 2000-ms (*p* = 0.026) tones. Formalin injection also decreased rats’ PSE compared with saline injection [*t*_(9)_ = −2.646, *p* = 0.027; Fig. 3b], indicating subjective overestimation of time. The average response latency to each tone duration took an inverted U shape (Fig. 3c). Response latency did not differ according to treatment, suggesting that physical activity was not affected by formalin injection. Formalin injection did not produce a significant difference in the SD [*t*_(9)_ = 0.135, *p* = 0.304; Fig. 3d], indicating no effect on temporal sensitivity. We also compared data from omitted trials performed after saline (average, 2.3 ± 1.0 trials) and formalin (average, 2.5 ± 0.5 trials) injections, and found no difference between treatments [*t*_(9)_ = 0.169, *p* = 0.869].

### Effects of SNL-induced neuropathic pain on interval timing behaviors

**Figure 4:**
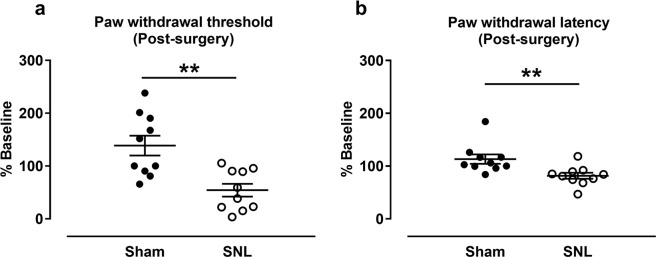
Results of the von-Frey and thermal withdrawal tests at 14 days after SNL. SNL surgery significant decreased the paw withdrawal threshold relative to baseline (**a**) and paw withdrawal latency relative to baseline (**b**) compared with the sham group. **p < 0.01 vs. sham group. *n* = 10/group. Results are shown as means ± SEM.

In 14 days after SNL surgery, paw withdrawal threshold [PWT, relative to the baseline, (Baseline: Sham group, 4.0 ± 0.5 g, SNL group: 5.7 ± 1.3 g; Day 14: Sham group, 4.0 ± 0.7 g, SNL group: 2.0 ± 0.1 g)] in the von Frey test was significantly lower in the SNL group than in the sham group [*t*_(18) _= 3.788, *p* = 0.001; Fig. 4a]. Paw withdrawal latency [PWL, relative to the baseline. (Baseline: Sham group: 13.7 ± 0.5 s, SNL group: 13.6 ± 0.7 s; Day 14: Sham group: 15.4 ± 1.1 s, SNL group, 10.8 ± 0.4 s)] in the thermal test was also significantly lower in the SNL group than in the sham group [*t*_(18) _= 2.999, *p* = 0.008; Fig. 4b]. These results confirmed that the rats had allodynia and thermal hyperalgesia 14 days after SNL surgery.

**Figure 5:**
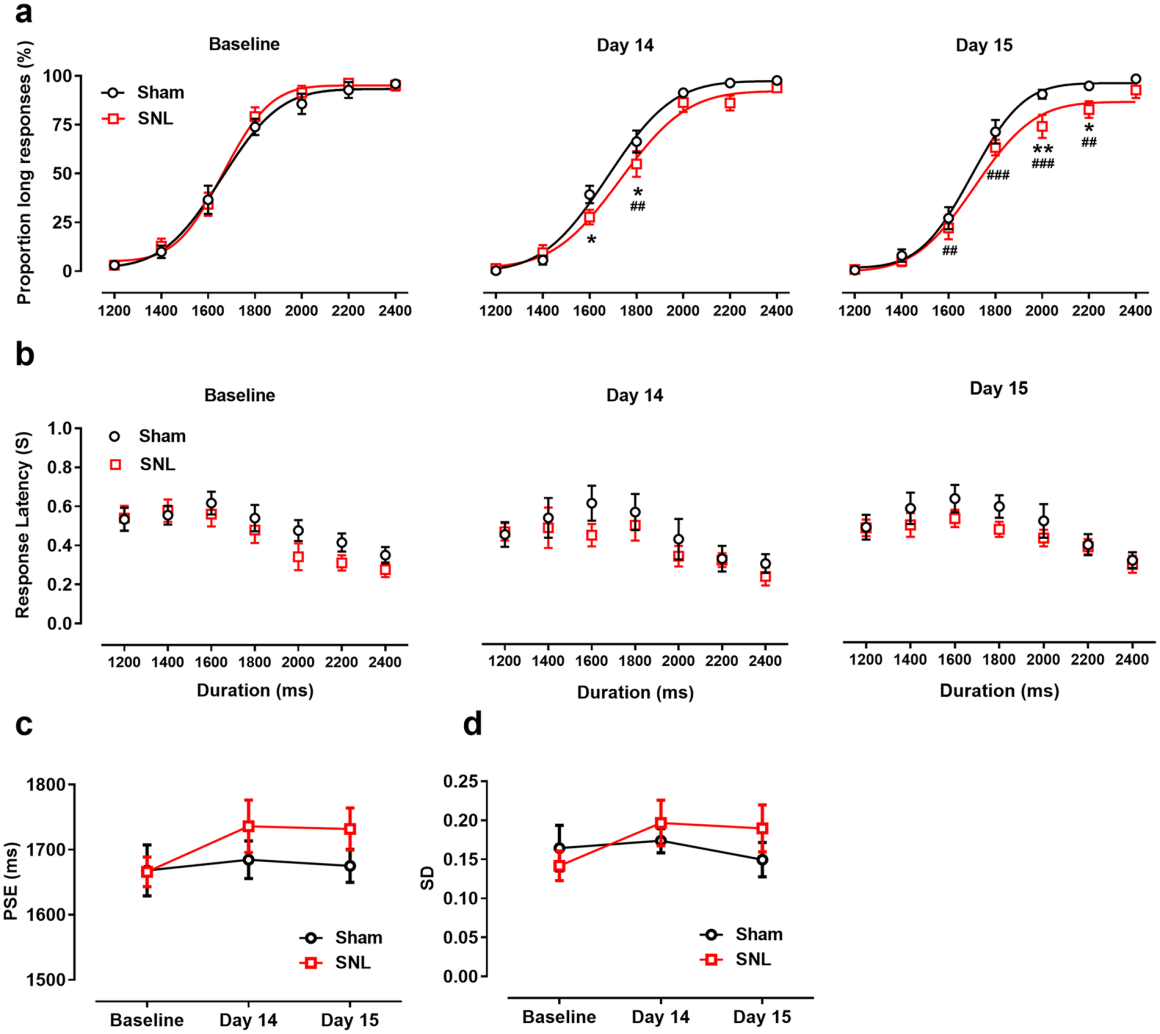
Response patterns in temporal bisection tasks [Proportion of long response (P_L_) by duration and psychometric fitting curves] at baseline and on days 14 and 15 after SNL surgery. SNL surgery significantly reduced the P_L_ in days 14 (middle panel) and days 15 (right panel) compared with the sham group and baseline (left panel) (**a**), but did not affect response latency in baseline (left panel) and days 14 (middle panel) and days 15 (right panel) (**b**). The point of subjective equality (PSE) (**c**) and standard deviations (SD) of fitting function (**d**) did not differ among the three sessions of the temporal bisection task. *p < 0.05, **p < 0.01 vs. sham group; ^##^*p* < 0.01, ^###^*p* < 0.001 vs. baseline; *n* = 10/grou*p*. Results are shown as means ± SEM.

Results from temporal bisection tasks performed before and day 14 and day 15 after surgery are presented in Fig. 5. The fitting curve explains most variation in these experimental data. The goodness of fit of the Gaussian function for individual rats was ≥0.87. After surgery, the fitted function for the SNL group shifted to the right and downward compared with those for the sham group and baseline. On days 14 and 15, the P_L_s to some tone durations were lower after surgery [interactive effect: *F*_(12, 216)_ = 2.468, *p* = 0.005; time effect: *F*_(1, 18)_ = 3.073, *p* = 0.059, Fig. 5a]. Specifically, on day 14, rats with SNL had lower P_L_s for the 1600-ms (*p* = 0.034) and 1800-ms (*p* = 0.030) tones compared with the sham group, and a lower P_L_ for the 1800-ms tone (*p* < 0.001) compared with baseline; on day 15, rats with SNL had lower P_L_s for the 2000-ms (*p* = 0.003) and 2200-ms (*p* = 0.043) tones compared with the sham group, and lower P_L_s for the 1600-ms (*p* = 0.009), 1800-ms (*p* < 0.001), and 2200-ms (*p* = 0.008) tones compared with baseline. Values for response latency during the temporal bisection task exhibited an inverted U shape (Fig. 5b). Response latency in the SNL group did not differ from that in the sham group or from baseline [interactive effect: *F*_(2, 36)_ = 0.184, *p* = 0.833; time effect: *F*_(2, 36)_ = 2.626, *p* = 0.086; group effect: *F*_(1, 18)_ = 0.048, *p* = 0.829].

Baseline and postoperative PSEs in the two groups did not differ significantly [interactive effect: *F*_(2, 36)_ = 0.36, *p* = 0.700; Fig. 5c]. In addition, the SD of the fitting function did not differ across the three test sessions [interactive effect: *F*_(2, 36)_ = 0.711, *p* = 0.498; Fig. 5d]. Average values from omitted trials did not differ from baseline (SNL group, 0.8 ± 0.2 trials; sham group, 0.9 ± 0.2 trials) to 14 days (SNL group, 1.3 ± 0.3 trials; sham group, 1.0 ± 0.15 trials) and 15 days (SNL group, 1.2 ± 0.2 trials; sham group, 1.0 ± 0.3 trials) after surgery [interactive effect: *F*_(2, 36)_ = 1, *p* = 0.377].

**Figure 6:**
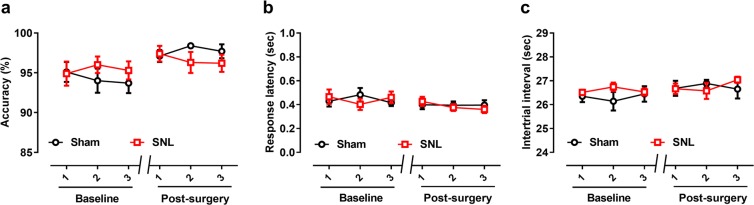
Comparison of response accuracy, the intertrial interval (ITI), and response latency in the last three training sessions before and three sessions after SNL surgery. (**a**) Before the test stage, the average response accuracy exceeded 90%, with no difference between groups. Response latency (**b**) and the ITI (**c**) did not differ between the SNL and sham groups. Results are shown as means ± SEM.

The two groups of rats showed no significant difference across six training sessions (three preoperative free-choice training sessions, three postoperative sessions) in response accuracy [interactive effect: *F*_(5, 90)_ = 1.227, *p* = 0.303; Fig. 6a], response latency [interactive effect: *F*_(5, 90)_ = 1.238, *p* = 0.298; Fig. 6b], or the ITI [interactive effect: *F*_(5, 90)_ = 0.913, *p* = 0.476; Fig. 6c]. These results indicate that SNL surgery did not affect the rats’ basic physical activity or time discrimination ability.

## Discussion

In this study, we used a temporal bisection task to assess rats’ time perception under formalin-induced acute pain and SNL-induced neuropathic pain. We found that acute inflammatory pain prolonged the estimation of time in the range of 1.2–2.4 s, whereas neuropathic pain exhibited a complex effect on time perception. Neuropathic pain lowered the P_L_s to the intermediate durations within this range, but not affected the PSE significantly. Neither pain type affected temporal sensitivity. These results suggest that the acute and neuropathic pain states have different effects on time perception.

Our results for formalin-induced acute pain are consistent with those of human studies, which have demonstrated that subjects overestimate stimulus duration under acute pain^19,20^. One explanation for this overestimation is acceleration of the internal clock mechanism, as conceptualized via the pacemaker-accumulator (PA) model. This model assumes that the internal clock generates “pulses” in the process of time perception, and that the estimation of time is based on the cumulative number of these pulses^1,41^. High arousal can accelerate pulse accumulation, leading to a subjectively longer experience^2^. Human subjects under experimental pain exhibit symptoms of high physiological arousal, such as increased heart rate and blood pressure^42,43^. Formalin-induced acute inflammatory pain, used widely in animal studies^44^, also induces a high-arousal state by facilitating the release of noradrenaline^45–48^. Increased heart and respiratory rates have been observed in rats after formalin injection^49,50^. Therefore, according to the PA model, the high arousal associated with formalin-induced pain contributes to the overestimation of time.

In contrast, after SNL surgery, rats’ P_L_s were lower when judging tone duration, with no change in the PSE or temporal sensitivity, in this study. Time perception is more complex in patients with chronic pain^21,22,51^. The impairment of arousal and attention caused by chronic pain may co-contribute to the effect of this type of pain on time perception. In the framework of the PA model, attention plays the key role of a “switch”^1,41^. When attention is distracted, the internal clock mechanism may not be activated, leading to the underestimation of temporal duration^10–12^. Chronic pain was also found to induce attentional bias toward pain-related stimuli, thereby influencing attention-related temporal dynamics^52^. Patients with neuropathic pain exhibit impairment of attentional resources^53,54^, and rats with such pain show poor performance in attentional tasks^55,56^. Abnormal activity in the medial prefrontal cortex (mPFC), which is related to attentional function, has also been identified in human patients^57^ and in animals^58^ with chronic pain. These results suggest that neuropathic pain’s impairment of attentional function may contributes to the change of time perception. In the neuropathic pain experiment, we did not measure time perception during the acute stage because rats needed to recover from SNL surgery for a few days. Therefore, this study did not compare the impact of acute and chronic pain on time perception in the same model. This is a limitation of this study.

Thus, whereas formalin injection induced acute pain (i.e., due to high arousal) leads to time overestimation, the SNL surgery induced chronic neuropathic pain has a complex effect on time perception. Different forms of chronic pain may be accompanied by different arousal states, with different effect on time perception. Our findings for neuropathic pain are not consistent with those of previous human studies of migraineurs^21,22,51^. These subjects may maintain high arousal states for some time, especially during pain attacks^59^. They exhibit abnormal temporal discrimination during migraine attacks, but normal discrimination during headache-free periods^24,25^. We thus consider that high arousal during migraine attacks contributes to time overestimation. On the contrary, neuropathic pain can lead to noradrenergic injury in rats^60^, reduce arousal, and thus fail to produce the prolongation of time estimation.

In addition, dopamine (DA) neurons in the midbrain may also be an underlying mechanism of the abnormal changes of time perception during chronic pain. It has been proved that DA system plays an important role in the process of time perception^61,62^. Soares *et al*. found that activation or inhibition of midbrain DA neurons was sufficient to slow down or speed up time estimation in mice, respectively^9^. Meanwhile, chronic pain may influence DA neurons in the ventral tegmental area (VTA). It has been demonstrated that partial sciatic nerve ligation induced neuropathic pain significantly decreased the proportion of activated DA neurons in the lateral VTA^63^. However, Fu *et al*., found that that spared nerve injury (SNI) can significantly increase the firing rate of VTA DA neurons^64^. These opposite effects may be due to the heterogeneity of DA neurons in VTA and the opposite functions of medial and lateral neurons in VTA^65^. Therefore, the activities of VTA-DA neurons may play a complex role in mediating the effect of chronic pain on time perception.

The lengthening effect of acute pain on time perception may have an evolutionary basis. Under acute pain, time overestimation caused by high arousal and concentration can reduce injury by leading to withdrawal from a (potentially) harmful environment^66^. Droit-Volet^67^ suggested that the dynamic perception of time involves the defensive system, with the temporal lengthening effect when facing a threat associated with a state of alertness, mobility, and readiness to act (fight back or run away). Therefore, acute pain–induced temporal lengthening may be associated with the organism’s preparation to avoid (even inevitable) pain. Chronic pain, in contrast, is caused by a different set of physiological and pathological changes^68^. Patients have characterized neuropathic pain as widespread and inexplicable, with pain attacks occurring seemingly without provocation^69^. In the absence of an obvious source of injury to avoid, temporal lengthening may not be important under chronic pain.

Acute pain can also cause distraction. Previous behavioral studies have shown that formalin injection significantly disrupts attentional tasks in rats, with increased response latency and omissions^16,17^. Acute inflammatory pain can alter neuronal activity in the mPFC in rats^18,70,71^. In our preliminary experiments, we injected rats with 5% formalin, which resulted in a significantly higher omission rate during the task (data not shown), suggesting that acute pain caused strong distraction. To complete the experiment smoothly, we chose to use 1% formalin, which may not be a sufficient concentration to disrupt the temporal bisection task. This formalin dose caused less nociceptive behavior and fewer omissions, suggesting the absence of strong distraction. Meanwhile, the cumulative time spent in paw lifting and licking after formalin injection during the task were significantly shorter than that without task. This phenomenon suggests that temporal bisection task distracted some attention resource from the formalin pain. According to the limited attentional capacity theory, the more attentional resources used by distraction, the less resources are available for perceiving pain^72^.

We also took measures to rule out the effects of other confusing factors. Chronic pain was found depress the activity of the mesolimbic dopamine system and lead to decreased reward-seeking motivation in animals^37^. To exclude this factor, we examined temporal bisection task trial omission. Neither neuropathic nor formalin-induced pain increased the omission rate, indicating that the animals maintained reward-seeking motivation during the temporal bisection task. In addition, pain may impair physical activity^73^ and perceptual accuracy^74^. In this study, we found that SNL surgery had no effect on response latency, the ITI, or response accuracy, and that response latency did not increase after formalin injection. These results indicate that the SNL surgery and formalin injection did not affect physical activity during the task. Furthermore, some studies have suggested that depression associated with chronic pain mediates changes in time perception in human patients^22,23^. Depressed patients exhibit poor sensitivity to long-duration stimuli, but normal sensitivity to short-duration stimuli^75,76^. Therefore, we performed open field and sucrose preference tests, but did not find significant depressive-like behavior in the rats after SNL surgery (Supplementary information Fig. S1). Some studies have observed depressive symptoms at 2 weeks^77^ or 4–6 weeks^64^ after spare nerve injury (SNI) surgery in rats. These studies used the same species of animal as our present study, but a different neuropathic pain model. Different pain models, pain duration and different laboratory environment may jointly affect the induction of depressive-like behavior. In the future, longer observation time or other model attempts, such as SNI, may be helpful to further explore the effect of pain depression comorbidity on time perception.

In conclusion, we found that formalin-induced and neuropathic pain have different effects on time perception. An important issue in the field of pain research is the manner in which acute pain translates into chronic pain^68^. Our research suggests that the neurological mechanisms associated with time perception are related to the occurrence of chronic pain. Most importantly, findings from this study lay a foundation for further research on the mechanism by which pain regulation affects time perception.

## Supplementary information

Supplementary information

## Data Availability

The experimental generated and analyzed during this study are available from the corresponding author on reasonable request.
