# Supplementary material for: Formalin-induced and neuropathic pain altered time estimation in a temporal bisection task in rats
Source: Sci Rep. 2019 Dec 10;9:18683. doi: 10.1038/s41598-019-55168-w (PMC6904569; doi:10.1038/s41598-019-55168-w)
Supplement: Supplementary file 1 — Supplementary information [file 41598_2019_55168_MOESM1_ESM.pdf]

Formalin-induced and neuropathic pain altered time estimation in a temporal bisection task in rats

Xinhe LIU, Ning WANG*, Jinyan WANG, Fei LUO

Supplementary information

We used the open field test (OFT) and the sucrose preference test (SPT) to examine the depressive-like behaviors after SNL surgery in rats. The OFT and SPT were performed once before the SNL and sham surgery as baseline; 16 days after the surgery, the OFT and SPT were performed again. Body weights were also measured before and after surgery.

Method:

The OFT was performed in an apparatus consists of a dark floor and a circular area (180 cm in diameter with a 50 cm-high wall), which located in a quiet environment with 2 dim lighting bulbs (40 w). Each rat was placed in this open field apparatus and free to explore this area for 5 min. The locomotor behavior and rearing behavior were recorded. The olfactory cues were washed with 75% ethanol at intervals between each test.

After the temporal bisection task, rats were deprived of water for 24 hours and familiar with 1% sucrose solution for 2 minutes before the sucrose preference test (SPT). The SPT was performed in a standard breed box. A bottle of plain water and a bottle of sucrose solution were put into the box simultaneously. Rats were free to drink for 1 h. The relative position of bottles was counterbalanced. Sucrose preference was defined as the percentage of sucrose water consumption on the whole liquid consumption, which could be calculated as:

$$\text{Sucrose preference} = [\text{sucrose solution consumption}/(\text{sucrose solution consumption} + \text{water consumption})] \times 100\%.$$

Two-way ANOVA was used for data analysis and $p < 0.05$ was considered to indicate significance. All data are expressed as means ± standard errors of the mean. The statistical analyses were conducted with Prism (version 5, GraphPad software, Inc) and STATISTICA (version 6, StatSoft.Inc) software.

Results

Results of depressive-like behaviors were presented in Fig. S1. The locomotor behavior, rearing behavior, sucrose preference, and body weights of SNL group did not have significant difference compared with these of sham group.

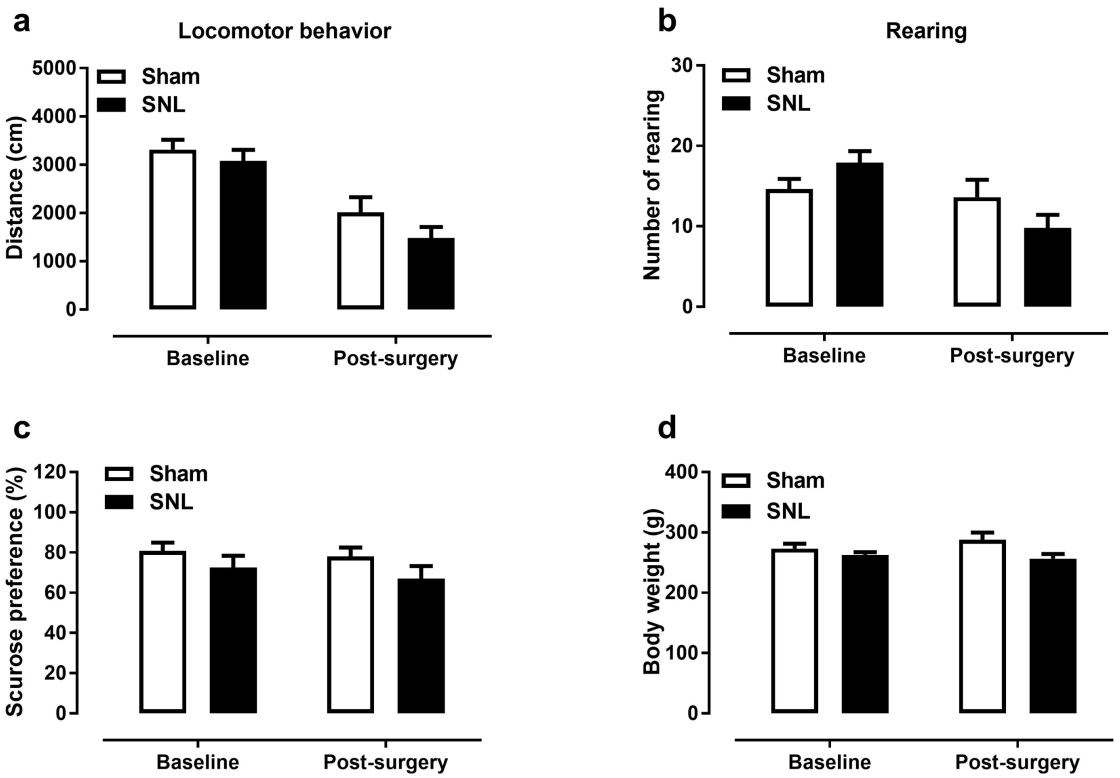

Fig. S1. The depressive-like behaviors after SNL surgery. (a) Locomotor behaviors; (b) Rearing behaviors; (c) Sucrose preference. (d) Body weights. N=10. Results are shown as means ± SEM.
